# Impact of Wall Material-to-Active Ratio in the Stability of Spray-Dried Ascorbic Acid Using Maltodextrin and Gum Arabic

**DOI:** 10.3390/molecules29153587

**Published:** 2024-07-30

**Authors:** Adeline Delaporte, Benoît Duchemin, Michel Grisel, Ecaterina Gore

**Affiliations:** 1 Université Le Havre Normandie, Normandie Univ, URCOM UR 3221, F-76600 Le Havre, France; adeline.delaporte@doct.univ-lehavre.fr (A.D.); michel.grisel@univ-lehavre.fr (M.G.); 2 Université Le Havre Normandie, Normandie Univ, CNRS, LOMC, F-76600 Le Havre, France; benoit.duchemin@univ-lehavre.fr

**Keywords:** encapsulation, spray-drying, vitamin C, maltodextrin, arabic gum, stability

## Abstract

Encapsulation revolutionizes industries through enhanced stability, controlled release, and targeted performance of active ingredients. The novel aspect of this study explores the impact of the wall material-to-active (WM:A) ratio on the stability of ascorbic acid (AA) encapsulated in a maltodextrin (MD) and gum arabic (GA) blend (2:1 *w/w*). Microparticles were spray-dried and analyzed using SEM, TGA, DSC, thermal stability, and antioxidant activity assessments. Stability tests under different conditions revealed that a higher WM:A ratio (7:1) improved the active stability and antioxidant activity during storage, highlighting its importance in the encapsulation process. SEM analysis confirmed particles with no cracks, and the particles demonstrated excellent thermal stability up to 200 °C with minimal degradation. These findings underscore the critical role of the WM:A ratio in determining the stability of encapsulated AA within a carbohydrate matrix, offering valuable insights for advancing encapsulation technologies.

## 1. Introduction

Vitamin C, also known as L-ascorbic acid (AA), is a naturally occurring water-soluble antioxidant. It plays an indispensable role in various physiological processes, including collagen synthesis, iron absorption, immune function enhancement, and the neutralization of harmful free radicals [1]. Despite its health advantages, the use of AA in food, pharmaceutical, and cosmetic industries presents considerable challenges. Exposure to light, oxygen, heat, alkaline pH, and the presence of transition metals can lead to the oxidation of AA. This process forms dehydroascorbic acid, which then undergoes hydrolysis to produce 2,3-diketogulonic acid, compromising its biological efficacy and bioavailability [2,3]. In aqueous solution, AA was found to form 2-furoic acid, 3-hydroxy-2-pyrone, and furfural, the latter being susceptible to combine with amino acids to form brown pigments [4]. However, describing all degradation phenomena is challenging, as it depends on many factors and conditions.

As a technique in which a bioactive compound is enclosed within a protective coating, microencapsulation has been increasingly used as an efficient method to deal with the instability of active compounds. Among other benefits, encapsulation protects the active compound from harmful external factors, thereby enhancing its stability [5,6]. Moreover, it provides controlled release properties, allowing for gradual release of the encapsulated material over time. These properties can benefit various applications, such as sustained release in pharmaceuticals and gradual release in cosmetic products [5,7].

A variety of techniques have been applied for the encapsulation of AA, such as hot-melt extrusion [8], layer–bilayer assembly [9], laser printing [10], and electrospinning [11], and among them, spray drying remains a popular and efficient method because of its cost effectiveness, operational ease, and suitability for large-scale production. Numerous factors influence the efficacy of the spray-drying process. These include the selection of wall material [7,12], size of the droplets in the initial emulsion [13], and spray-drying parameters such as gas flow rate, liquid flow rate, and air inlet and outlet temperatures [14]. Encapsulation efficiency can be affected by the interaction between the chosen wall materials and the active compound and the ratio of the wall material to the active compound [15]. For successful encapsulation of the active compound, it is essential for the wall material to possess suitable properties. These include compatibility with the active ingredient, film-forming capabilities, low viscosity at high concentrations, easy atomization during spray drying, and excellent solubility in the solvent system selected for encapsulation. As no single wall material can fulfill all these criteria, they are often combined to achieve a balance of desired characteristics and performance [15].

To date, encapsulation of AA has been studied using various wall materials, such as starches [16,17], natural galactomannan [18], sodium alginate [19], gum arabic [20], maltodextrin [21], and combinations [17,22]. However, only a few studies have examined the stability of AA particles during storage. While Hoyos-Leyva et al. (2018) and Palma-Rodríguez et al. (2018) observed 30% remaining AA after four weeks at 55 °C with 72% and 50% relative humidity, respectively, they did not investigate the impact of the WM:A ratio on retention [16,17].

Maltodextrin (MD), a starch-derived polysaccharide, and gum arabic (GA), a natural gum harvested from the acacia tree, are frequently used for encapsulation through the spray-drying process. It is obvious that both MD and GA substances are valued for their excellent film-forming properties and water solubility, providing a protective shell around the active ingredient and enhancing its temperature and oxidation stability [12]. Previous research has demonstrated the effective encapsulation of various active compounds, such as vitamins [23,24], phenolic compounds [25,26,27], and oils [28] using these two biopolymers, thus enhancing their stability. There is still a limited understanding of encapsulating water-soluble active ingredients using this combination of biopolymers. Furthermore, there is insufficient comprehensive information on how changes in the ratio of wall material to active ingredient affect the physicochemical properties and stability of spray-dried particles. The present study aimed to investigate the effect of the wall material-to-active (WM:A) ratio on the characteristics and stability of AA encapsulated in a carbohydrate matrix composed of a blend of MD and GA particles produced by spray drying. Keeping the total solid concentration and MD:GA ratio constant, this study investigated how this ratio affects the encapsulation efficiency, morphological characteristics, and thermal stability as well as the retention of ascorbic acid and its antioxidant activity under various storage conditions.

## 2. Results and Discussion

### 2.1. Microparticles Production and Characterization

MDGA-AA microparticles were produced by spray drying and prepared from solutions containing different WM:A mass ratios. Thus, its impact on the particle production, physicochemical properties, and stability of the particles was evaluated. The corresponding data are presented in Table 1.

#### 2.1.1. Preparation of the Solutions

The product yield varied from 46.3 to 55.6%, with no significant differences when the WM:A ratio increased. Yield is conditioned by the drying process’s efficacy and the particles’ stickiness, inducing potential loss during the spray-drying process. In fact, the stickier particles adhere to the wall of the drying chamber and cannot be collected, resulting in a small amount of product recovered. Similar results have been reported for the encapsulation of AA and macadamia oil using carbohydrates [29,30]. Indeed, Nizori et al. (2020) reported that the most concentrated particles did not dry properly and self-aggregated during the process, consistent with the slight decrease observed in (Table 1) for the highest loading contents [29].

#### 2.1.2. Active Loading and Encapsulation Efficiency

UV/vis characterization allows access to various types of information, such as active loading, encapsulation efficiency, and radical scavenging activity (Table 1). Changing the WM:A ratio directly impacts the active loading of microparticles, increasing from 15.32 to 40.6% when the ratio decreases from 7:1 to 2:1, respectively. Bhandari et al. (1992) reported a similar finding, showing an increase in the volatile concentration in the powder when the initial quantity of volatile compounds was increased [31].

As shown in Table 1, the encapsulation efficiency significantly increased from 55.1% to 68.2% as the ratio increased from 3:1 to 7:1. As it is linked to the mass of microparticles recovered, the results followed the same trend as the yield. Previous studies have reported similar results for encapsulating fish oil, flaxseed, and volatiles [29,31,32]. Lower ratios may result in an inadequate quantity of wall material, leading to reduced encapsulation efficiency [29,32]. 

#### 2.1.3. Antioxidant Activity of Encapsulated AA

The powders’ antioxidant activity was assessed using DPPH and UV/vis, with the data presented as % scavenging activity per mg of powder in Table 1. As expected, the 2:1 ratio exhibited the highest scavenging activity, while the 7:1 ratio showed the lowest due to its lower antioxidant concentration (40.60% and 15.32%, respectively). A strong positive correlation (r = 0.994, *p* < 0.05) was found between these two parameters. Loading more antioxidants into each microparticle reduces the number of microparticles needed to achieve the desired antioxidant activity in the final product. Depending on their application, this could lead to cost savings and enhanced product effectiveness.

### 2.2. Hygroscopicity

The spray-dried microparticles were placed in an environmental chamber at 25 °C and 80% RH. After one week, their hygroscopicity (i.e., capacity to absorb environmental moisture) was found to range from 12.7% to 19.3%, increasing with the WM:A ratio, as shown in Table 1. A similar behavior was reported by de Barros Fernandes et al. (2014) for the encapsulation of rosemary essential oil [33], where the highest hygroscopicity was reached for the highest concentration in the wall material. In addition, similar values of hygroscopicity were observed by Silva et al. (2013) (17.6%) [34], Frascareli et al. (2012) (13.7–17.9%) [35], and de Barros Fernandes et al. (2014) (9.3–13.9%) [33]. Thus, increasing the wall material concentration results in the accelerated formation of the particle crust during drying, consequently preventing the water from evaporating [32]. Particle size may also play a key role. In a study by Kurozawa et al. (2009), hygroscopicity increased with a decrease in the size of GA particles [36]. Reducing the particle size results in an increase in the overall contact surface area and thus in moisture absorption.

### 2.3. Morphology and Particle Size

**Figure 1:**
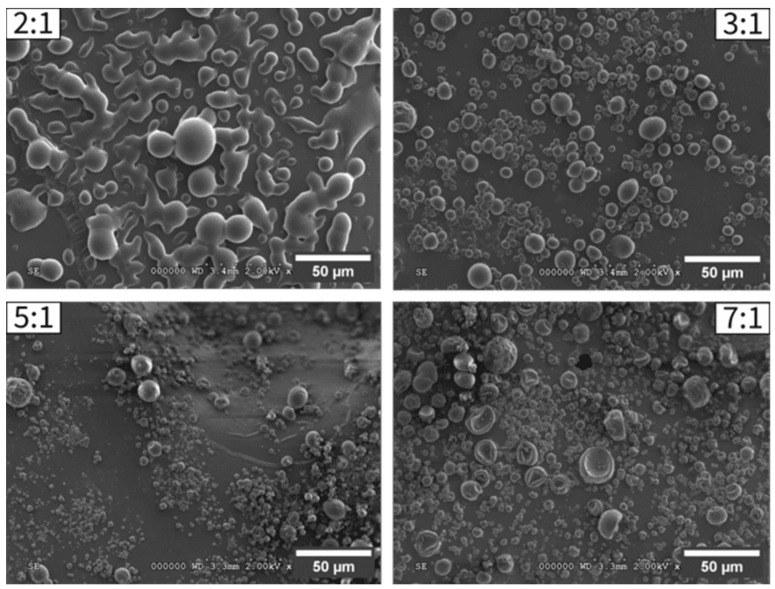
Microstructures of MDGA-AA powders produced with different wall material/active (WM:A) ratios (*w*/*w*): 2:1, 3:1, 5:1, and 7:1.

Microparticles produced at WM:A ratios of 3:1, 5:1, and 7:1 presented a spherical shape, whereas those at a 2:1 ratio appeared fused and clumped with irregular shapes, as indicated by larger particle sizes with high variability (Table S1). All particles were evidenced as free of cracks, limiting the permeability to gas and suggesting better retention of the encapsulated active over time. Furthermore, they all displayed a broad size distribution, which was confirmed by calculating the span (Table S1), which is typical for spray-dried particles. Indeed, similar results were obtained by Tonon et al. (2011) and Oliveira et al. (2005), with a span value of approximately 1.99–2.65; the larger the value, the wider the size distribution [32,37]. When the proportion of wall material increased, more individual particles were produced with a visible decrease in size (Figure 1): D_50_ decreased from 21.7 µm at a 2:1 ratio to 7.1 µm for a 7:1 ratio. Nizori et al. (2020) and Rodklongtan et al. (2022) also reported an agglomeration of particles at higher AA concentrations, as their drying time was too short to ensure the formation of individual particles [29,38].

### 2.4. Differential Scanning Calorimetry

**Figure 2:**
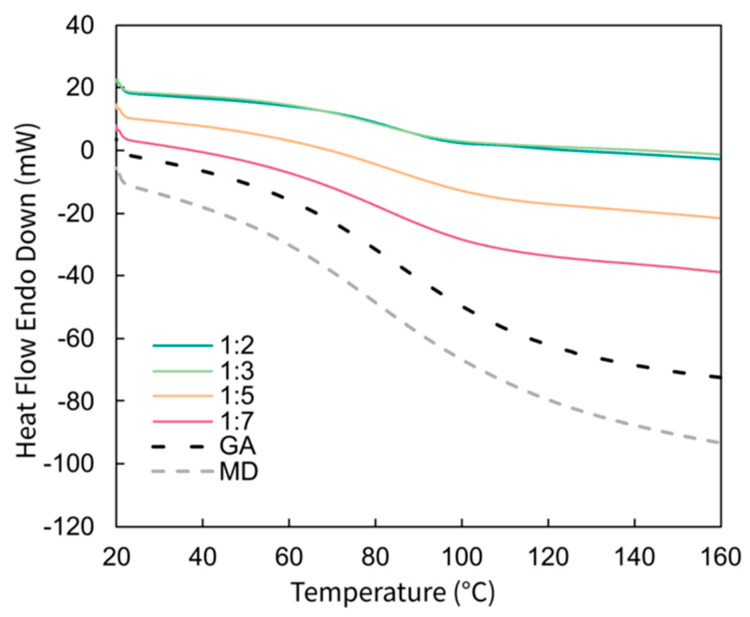
DSC thermograms of pure maltodextrin (MD), gum arabic (GA), and MDGA-AA particles depending on the ratio WM:A (*w*/*w*).

Differential scanning calorimetry (DSC) analyses of the wall materials and produced microparticles were conducted to evaluate their thermal properties from 20 to 160 °C (Figure 2). In particular, glass transition temperatures (Tg) were determined (Table 1). As reported by de Barros Fernandes et al. (2014), the stability of particles during processing or storage can be evaluated by measuring their Tg values [39]. If the latter is too low, the particles may undergo structural changes, which can cause early release or accelerated degradation of the active material. The wall materials MD and GA exhibited Tg of 86.1 °C and 91.1 °C, respectively. For the MDGA-AA microparticles, the WM:A ratio had no significant impact on the calculated Tg between 77 and 81 °C. Rodklongtan et al. (2022) reported a different trend with a decrease in Tg with an increase in encapsulated AA concentration. This was attributed to the small size of the vitamin, which increased the lactose chain mobility [38]. However, Barra et al. (2019) encapsulated AA with sodium alginate on the one hand and GA on the other, and a positive impact on Tg was only noticed in the second system [22]. This indicates that the components, either wall materials or actives, have a complex impact on the crystallinity of the produced microparticles. Considering that GA and MD are the main contributors to the Tg and that the MD:GA ratio was maintained constant at 2:1 throughout all produced microparticles, this might explain the absence of impacts on the measured Tg. MDGA-AA microparticles produced in this study can all be stored at temperatures up to 50 °C with limited degradation and without experiencing stickiness issues, the latter occurring at 10 °C above Tg [40].

### 2.5. Thermogravimetric Analysis (TGA)

**Figure 3:**
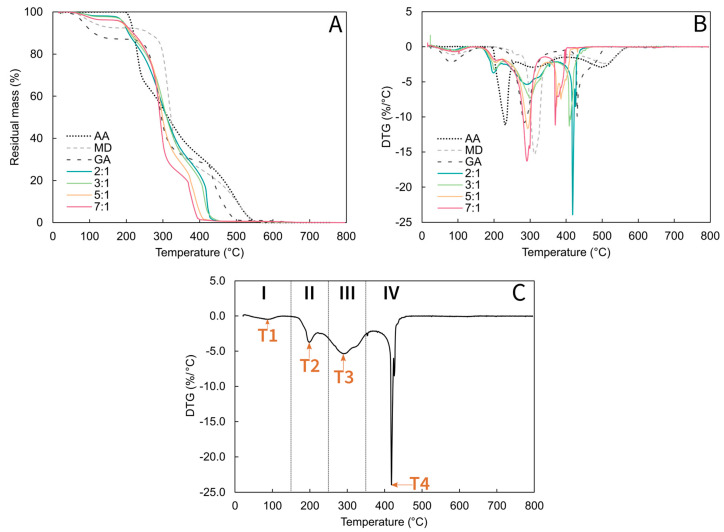
Thermal degradation profile of maltodextrin (MD), gum arabic (GA), ascorbic acid (AA), and MDGA-AA particles (**A**) and the associated differential curves (**B**) and example of analysis (**C**). I–IV correspond to the four temperature steps the loss mass occurs.

The thermo-oxidative stability of MDGA-AA microparticles was evaluated by performing TGA analyses under an air atmosphere from 25 to 800 °C. Corresponding thermograms and their differential curves (DTG) are presented in Figure 3A and 3B, respectively; also, degradation steps identified by recording DTG peak values with their associated mass loss are gathered in Table 2. All samples were characterized by four steps that were identified to analyze the mass loss: 25–150 °C, 150–250 °C, 250–350 °C, and 350–800 °C, which are referred to as steps I, II, III, and IV, respectively (Figure 3C).

First, the degradation profiles of the pure raw materials were assessed. Wall materials presented a three-step degradation process: The first step occurred at temperatures around 100 °C with a mass loss of 7% for MD and 13% for GA, which is associated with water desorption. The second step took place around 300 °C, with a mass loss of 58% for MD and 52% for GA, which is related to the polysaccharides decomposition [22,28]. Then, the last step occurred at around 500 °C for MD and 430 °C for GA. According to the literature, AA started its decomposition around 190 °C to reach a maximum rate of 230 °C with 33% mass loss, followed by two degradation steps around 310 °C and 500 °C [41]. Considering MDGA-AA microparticles, a shift in degradation peaks (T2) towards higher temperatures was observed when the WM:A ratio was increased. As this stage involves the degradation of AA, such a shift may be associated with its initial concentration before the spray-drying process. Similar results were reported by Barra et al. (2019) for the encapsulation of AA in combination with sodium alginate and GA [22]. All the produced microparticles exhibited improved thermal stability up to 200 °C, which allows their use in high thermal processes with limited loss.

### 2.6. Retention of Encapsulated AA under Various Storage Conditions (UV/vis)

**Figure 4:**
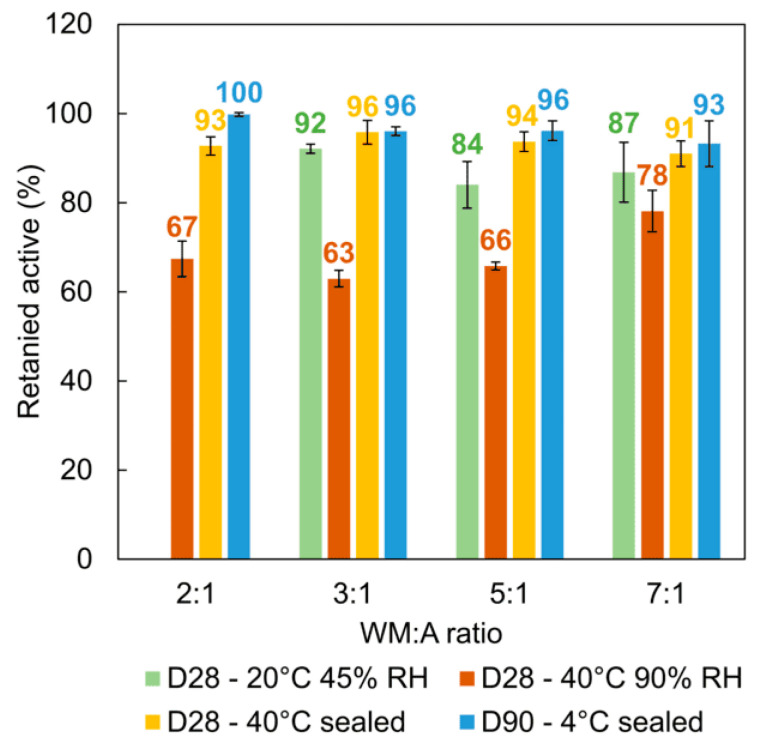
Concentration of retained active after 4 weeks at 20 °C–45% RH and 40 °C–90% RH under air exposure and after 3 months at 40 °C and 4 °C in sealed containers. Error bars represent the standard deviation values.

The ability of microparticles to retain the active over time was assessed by placing them in unsealed containers placed in a climatic chamber under two distinct conditions: 20 °C and 45% RH and 40 °C and 90% RH, respectively; additional hermetically sealed containers were stored at 4 °C and 40 °C, respectively. These conditions were selected in order to visualize the potential effects of exposure to air and humidity and simulate everyday storage situations. The concentration of the retained AA in the microparticles was determined by UV/vis spectroscopy (Figure 4). Additionally, HPLC-MS analysis was also performed, which demonstrated that the UV/vis analysis results were not influenced by any degradation products (Figure S3).

First, particles stored at 20 °C and 45% RH sharply agglomerated and became more compact. Unexpectedly, the most compact particles were produced at a WM:A ratio of 2:1, forming a single block of strongly agglomerated particles and thus preventing any measurement. After four weeks at 40 °C and 90% RH, all samples agglomerated due to swelling under excessive environmental humidity. In addition, browning of the microparticles was observed, which may be due to their degradation by the Maillard reaction. Indeed, initially described as involving amino acids and reducing sugars, studies have revealed that AA and its degradation products can be involved in the Maillard reaction [42]. Hence, the protein fractions of GA, AA, and their degradation products may be responsible for this browning [43,44]. Nevertheless, no browning was observed at 40 °C in the absence of air exposure. 

Under ambient conditions at 20 °C and 45% RH, between 9% and 16% AA was lost, while raising the temperature and relative humidity resulted in increased active degradation. Similar observations were reported by Righetto and Netto (2006) for the encapsulation of cherry juice [44] and by Finotelli and Rocha-Leão (2005) employing MD and capsules for AA encapsulation [21]. Nonetheless, the degradation of AA was limited at a higher WM concentration, with approximately 78% of the initial AA content remaining at a WM:A ratio of 7:1. The sensitivity of encapsulated AA to high relative humidity was also reported by Hoyos-Leyva et al. (2018) using taro starch as the wall material [16]. Thus, this observation highlights the enhanced effect achieved under these conditions. 

The retention of MDGA-AA under storage conditions at 40 °C, without exposure to air, exhibited higher levels than the exposed samples. Furthermore, these results were comparable to those obtained for samples stored at 4 °C for three months. Thus, storing these microparticles in hermetically sealed containers can efficiently prevent active loss, particularly at low temperatures. Rodklongtan et al. (2022) also reported excellent stability with more than 95% retention after two months at 4 °C using lactose as a wall material [38].

In summary, considering all storage conditions studied, encapsulated AA showed good retention despite its sensitivity to temperature, oxygen, and humidity.

### 2.7. Long-Term Stability—40 °C/90% RH

#### 2.7.1. Effect of Storage on Microparticles and Pure AA

Approximately 0.1 g MDGA-AA particles and pure active was placed in open containers at 40 °C and 90% RH to evaluate the impact of the storage conditions on their macrostructure and weight. 

After a few days of storage, all MDGA-AA particles agglomerated to form a gel-like substance. Consistent with previous findings, the generated particles demonstrated a hygroscopic nature that can induce morphological alterations, affecting well-defined individual particles into gel through a process of coalescence. Wang et al. (2019) reported similar observations for protein particles produced by spray drying [38,45]. A mass gain between 25 and 29% was measured for MDGA-AA particles after the first week, with no WM:A ratio influence. A maximum was reached for three weeks after the powders’ weight decreased until stabilizing. Concerning AA, minor changes were observed until the third week, after which a mass change occurred, reaching almost 23% after 9 weeks (Figure S2). 

Considering the overall results, particles produced at ratios of 2:1 and 3:1 seemed to be the most impacted, with the highest variability over time and an increase of +7% and +2% wt. Such diversity may be explained by their morphology, particle size, porosity, and chemical composition [45]. 

#### 2.7.2. Retention of AA during Storage

**Figure 5:**
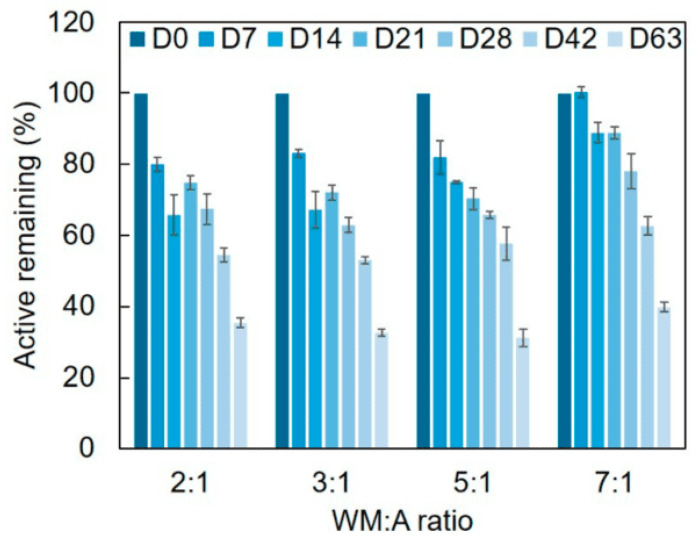
Concentration of remaining encapsulated over 9 weeks at 40 °C 90% RH. Error bars represent the standard deviation values.

The remaining concentration of active inside MDGA-AA particles placed at 40 °C 90% RH was evaluated over 9 weeks (Figure 5).

It was evident that all produced particles shared a similar behavior, with a decrease in AA content values over the entire storage period. Surprisingly, all microparticles showed a sharp loss of active content, except for the WM:A 7:1 ratio, which demonstrated much greater stability (*p* < 0.05), achieving the same level of degradation after four weeks. Nevertheless, approximately 31–40% of the AA remained encapsulated by the end of the nine-week period, with the highest results attributed to the highest WM:A ratio. Two-way ANOVA showed that the ratio and time significantly affected (*p* < 0.05) the stability of encapsulated AA. A similar tendency was observed by Palma-Rodríguez et al. (2018) using modified starch and MD as wall materials [17].

#### 2.7.3. Impact of Storage on Antioxidant Activity of Encapsulated AA

**Figure 6:**
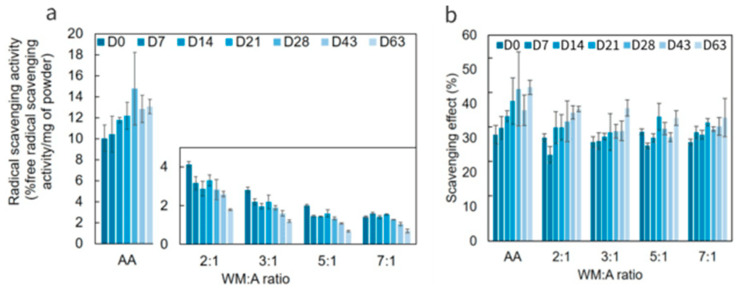
Radical scavenging activity of MDGA-AA particles according to the wall materials/active (WM:A) ratio (**a**) and at 0.3 g/L of ascorbic acid (AA) equivalent (**b**). Error bars represent the standard deviation values.

According to the procedure described in Section 3, the antioxidant activity of the encapsulated AA was assessed at 40 °C and 90% RH for nine weeks. Results are represented as percentage of free radical scavenging/mg of powder in Figure 6a and as %scavenging activity at constant AA concentration (Figure 6b) for pure and encapsulated AA.

As clearly visible in Figure 6, the antioxidant activity of MDGA-AA particles showed a significant decrease with increasing storage time, correlated with the remaining concentration of encapsulated active. Hu et al. (2018) also identified a positive correlation between the microcapsules active content and their antioxidant activities [46]. Among the others, the WM:A 7:1 ratio was identified as the most effective, with 49% of the antioxidant activity remaining, which is linked to its good retention capacity, as evidenced in Section 2.7.2. Considering pure AA, a significant increase in activity (+30%) was noticed after 63 days. Indeed, AA is known to be highly sensitive to both temperature, oxygen, and humidity. Consequently, it undergoes degradation reactions, leading to the formation of furan derivatives [2], with the corresponding potential antioxidant activity thought to contribute to the improvement of the activity of pure AA during storage [47,48]. Therefore, the loss of active content overcame the possible presence of degradation products, as no similar behavior was observed in the case of MDGA-AA particles. 

Nevertheless, all samples demonstrated increased activity over time when the results were analyzed regarding constant AA concentration (Figure 6b). Because there was visible browning of the samples, the Maillard reaction products could explain this phenomenon [49]. Carneiro et al. (2013) reported similar results when GA was used as a wall material, as its protein fraction promoted the Maillard reaction [12]. Consequently, the concentration of these products may rise over time, which may be responsible for the observed increase in activity.

## 3. Materials and Methods

### 3.1. Material

L-ascorbic acid ≥ 99% (AA) (batch MKCQ6208, lot 1003359829, Sigma-Aldrich, St. Quentin Fallavier, France) was used as active material (A). Maltodextrin 16DE (MD) (batch C*DRY MD 01915, lot 02267444, Caldic Ingredients, Villepinte, France) and gum arabic KLTA-UHF (GA) lot 3930898 (Kerry Group, Zwijndrecht, The Netherlands) were used as wall materials. Anhydrous sodium acetate (purity ≥ 99%), 2,2-diphenyl-1-picrylhydrazyl, 95% (DPPH•) (lot 044150.03), glacial acetic acid (≥99.5%), and pure methanol (MeOH) were purchased from Fisher Scientific (Waltham, MA, USA); all were used without further purification.

### 3.2. Preparation of MDGA-AA Microparticles

#### 3.2.1. Preparation of the Solutions

The wall materials (WM) MD and GA were blended together at an MD:GA ratio of 2:1 (*w/w*) and dissolved in distilled water at room temperature under the mechanical stirring of a VMI Turbotest (Turbotest, VMI-mixing, La-Roche-sur-Yon, France) equipped with a deflocculator turbine (diameter of 35 mm) at 1300 rpm for 10 min. The wall material solutions were prepared one day before emulsification and kept overnight at 4 °C to ensure full polymer hydration. Shortly before spray drying, AA powder was gradually added to the wall material solution under mechanical stirring at 8000 rpm for 2 min at room temperature using a rotor-stator blender T25 digital ultra-turrax equipped with turbine S25 N-25F (IKA, Freiburg, Germany). Four different wall materials/active (WM:A) ratios were investigated, namely 2:1, 3:1, 5:1, and 7:1 (*w*/*w*). The total solids content (active and wall materials) was fixed at 15% (*w*/*w*).

#### 3.2.2. Experimental Conditions—Spray-Drying Process

Spray drying was carried out using a BÜCHI B-290 (Flawil, Switzerland) with a 0.7 mm nozzle. The solutions were fed into the drying chamber by using a peristaltic pump at a flow rate of 9 mL/min (30%). The inlet and outlet air temperatures were maintained at 155 ± 1 and 75 ± 1 °C, respectively. The air pressure, airflow, and aspiration rate were set at 5–6 bar, 667 L·h^−1^, and 100% (35 m^3^·h^−1^), respectively. The dried powder was collected and stored in closed containers at 4 °C until further analysis. The product yield was calculated as the ratio between the dry mass of the recovered powder at the end of the process to the mass of the solid in the initial solution.

### 3.3. Spray-Dried Powder Characterization

#### 3.3.1. Active Loading and Encapsulation Efficiency

The microparticles were dissolved in deionized water at 1 g·L^−1^ and diluted (50 times) with 0.1 M acetate buffer pH 5 to fit the calibration curve. Analyses were performed in triplicate using a UV/vis spectrophotometer Shimadzu UV-1800 (Shimadzu, Duisburg, Germany) (λ = 265 nm). A calibration curve was developed in 0.1 M acetate buffer pH 5 to quantify the AA. An example of a UV/vis spectrum of MDGA-AA microparticles is provided in Figure S1, showing the absorption peak of AA at 265 nm.

The encapsulation efficiency (EE%) was calculated using Equation (1):

(1) $$EE\left(\%\right)=\frac{Active\;loading\;\left(\%\right)\;\times \;powder\;recovered\;\left(g\right)}{Ascorbic\;acid\;initially\;added\;\left(g\right)}\;.$$

The term “active loading” refers to the concentration of AA within dry particles, while the term “powder recovered” indicates the amount of microparticles obtained following the spray-drying process.

#### 3.3.2. 2,2-Diphenyl-1-Picrylhydrazyl (DPPH) Radical Scavenging Activity

The free radical scavenging activity of MDGA-AA microparticles was determined according to the procedure described by Brand-Williams et al. (1995) [50]. All microparticles were solubilized in deionized water and diluted in MeOH to a final concentration of 0.006 g·L^−1^. An aliquot (1 mL) was added to 2 mL of DPPH• 0.0375 g·L^−1^ in MeOH prepared daily. The absorbance at 515 nm was recorded after 5 min. The absorbance of the control was measured by replacing the sample with MeOH. The DPPH radical scavenging activity of the samples was calculated as follows (Equation (2)):

(2) $$DPPH\;radical\;scavenging\;activity\;\left(\%\right)\;=\;\frac{the\;absorbance\;of\;control\;-\;absorbance\;of\;sample}{absorbance\;of\;control}\;.$$

#### 3.3.3. Hygroscopicity

Powder hygroscopicity was determined according to the method adapted from Cai and Corke (2000) [51]. MDGA-AA microparticles (approximately 0.5 g) were weighed in Petri dishes and stored at 25 °C and 80% relative humidity (RH) in an environmental chamber (Memmert, Schwabach, Germany). The samples were weighed after one week, and the hygroscopicity was expressed as adsorbed moisture per 100 g of samples (g/100 g).

#### 3.3.4. Particle Morphology and Size Distribution

The surface morphology of MDGA-AA microparticles was visualized using a Hitachi S-3000 N field emission scanning electron microscope (Hitachi-HT, Tokyo, Japan), using magnification from ×500 to ×2000 and a working distance of 3.4 mm. Before the SEM analysis, all samples were carbon-coated using a JEC-530 apparatus (JEOL, Tokyo, Japan).

The particle size distribution was determined by laser granulometry using a Shimadzu SALD 7500 Nanoparticle size analyzer (Shimadzu, Kyoto, Japan) and Wing SALD II Version 3.1.0. The microparticles were first dispersed in isopropanol (Acros Organics, Geel, Belgium), vortexed for a few seconds to limit self-aggregation, and stirred during the analysis. Samples were analyzed by triplicate, and the results were expressed as average values of D [10], D [50], and D [90]. The uniformity and dispersity of the microparticles were represented by the span value, which was calculated using Equation (3):

(3) $$Span=\frac{\left(D[90]-D[10]\right)}{D[50]}\;.$$

A value close to zero indicates a monodisperse size distribution, whereas the higher the value, the wider the size distribution.

#### 3.3.5. Differential Scanning Calorimetry

Differential scanning calorimetry (DSC 8000, Perkin-Elmer, Waltham, MA, USA) was used to determine the glass transition temperature (Tg) of the spray-dried microparticles using the extrapolated half-ΔCp extrapolated method at half the extrapolated change in specific heat (ΔCp) between the glassy state and the rubbery state. Approximately 5 mg of the sample was weighed into an aluminum pan with holes under nitrogen gas at 50 mL·min^−1^. The curves were obtained by analyzing the samples at 10 °C·min^−1^ and a temperature range of 20–160 °C. Calibration was performed using indium and an empty pan was used as a reference.

#### 3.3.6. Thermal Gravimetric Analyses (TGA)/Derivative Thermogravimetric Analyses (DTG)

The thermal properties of the MDGA-AA powders (5–10 mg) were measured using a Setsys TGA 1200, SETARAM (Caluire-et-Cuire, France) apparatus from 25 to 800 °C under an air atmosphere. Moisture content was determined by measuring the decrease in weight at a temperature of 150 °C.

### 3.4. Storage Stability of Microparticles

**Figure 7:**
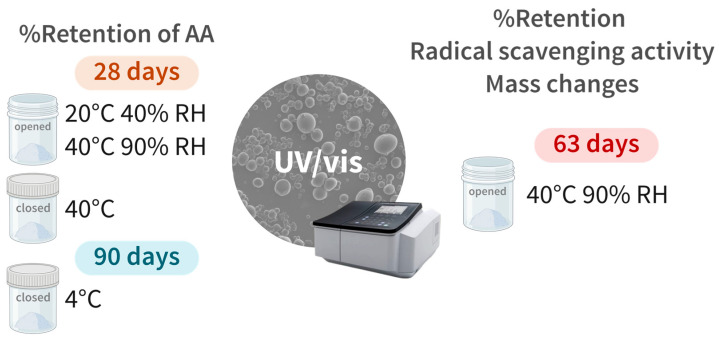
Selected storage conditions and evaluated parameters for the evaluation of the stability of ascorbic acid microparticles (MDGA-AA).

Selected storage conditions and evaluated parameters of the study are summarized in Figure 7.

#### 3.4.1. Short-Term Stability

MDGA-AA microparticles were stored in open vials at 40 °C and 90% RH and 20 °C and 45% RH in an environmental chamber (Memmert, Schwabach, Germany) and in closed vials at 40 °C for one month and at 4 °C for 3 months. Active loading was determined according to the method described in Section 3.3.1.

#### 3.4.2. Long-Term Stability

Previous samples stored in open vials at 40 °C and 90% RH were analyzed by UV/vis spectroscopy over 9 weeks following Section 3.3.1 (active loading and encapsulation efficiency) and Section 3.3.2 (DPPH radical scavenging activity). Mass changes were followed in these conditions by weighing 0.1 g of powder in vials with three replicates for each ratio and pure AA.

### 3.5. Statistical Analyses

All analyses were conducted at least by duplicate. Results are presented as the mean ± standard deviation (SD). The collected data were analyzed by one-way or two-way ANOVA and compared with Tukey’s HSD test at a confidence level of 95% using the XLSTAT software (Version 2012.1.01, Addinsoft, Paris, France). Pearson’s correlation coefficients were also determined using this software.

## 4. Conclusions

The study extensively explored ascorbic acid encapsulation through spray drying using polysaccharide-based materials, focusing on the impact of the WM:A ratio on MDGA-AA microparticle characteristics and stability. Increasing this ratio up to 7:1 (*w*/*w*) positively affected encapsulation efficiency and led to smaller particle sizes, suggesting improved performance during the spray-drying process. The microparticles exhibited smooth shapes without cracks, indicating limited gas permeability and enhanced active ingredient retention over time. They also showed excellent thermal stability at temperatures up to 200 °C and remarkable stability even under extreme storage conditions such as 40 °C and 90% relative humidity. Under these conditions, approximately 40% of the active compound remained encapsulated after two months, with about 49% of initial antioxidant activity retained. However, browning due to Maillard reaction was observed during storage at extreme conditions. The WM:A ratio represents a key parameter for producing highly stable AA particles with significant potential for effective encapsulation and sustained antioxidant activity in functional foods or pharmaceutical/cosmetic products, offering significantly longer shelf life. Therefore, further studies must be carried to evaluate the release of AA in various media and to prevent Maillard reaction from occurring during storage.

## Figures and Tables

**Table 1:** Characteristics of MDGA-AA particles according to wall material/active ratio (*w/w*). ^a,b,c,d^ values with different letters in the same column differ significantly (*p* < 0.05).

Ratio WM:A (*w*/*w*)	Yield (%)	Active Loading (%)	Encapsulation Efficiency (%)	Radical Scavenging Activity	Initial Moisture Content (%)	%Hygroscopicity	Tg (°C)
				(%Scavenging Activity/mg of Powder)			
2:1	48.0 ± 0.7 ^ab^	40.6 ± 1.6 ^a^	58.5 ± 1.6 ^a^	4.1 ± 0.2 ^a^	1.9 ± 0.0 ^a^	12.7 ± 0.2 ^a^	81.1 ± 3.2 ^a^
3:1	46.3 ± 0.9 ^b^	29.8 ± 1.4 ^b^	55.1 ± 2.2 ^a^	2.8 ± 0.1 ^b^	2.4 ± 0.1 ^b^	15.4 ± 0.4 ^b^	77.6 ± 1.4 ^a^
5:1	50.2 ± 5.7 ^ab^	20.1 ± 1.1 ^c^	64.1 ± 7.0 ^a^	2.0 ± 0.1 ^c^	3.6 ± 0.1 ^c^	18.4 ± 0.0 ^c^	79.6 ± 0.2 ^a^
7:1	55.6 ± 3.1 ^a^	15.3 ± 0.3 ^d^	68.2 ± 4.8 ^b^	1.4 ± 0.0 ^d^	3.9 ± 0.1 ^c^	19.3 ± 0.0 ^c^	80.7 ± 0.9 ^a^

**Table 2:** Degradation steps and associated mass loss of ascorbic acid (AA), maltodextrin (MD), gum arabic (GA), MDGA, and MDGA-AA particles.

Sample	Degradation Step (°C)				Mass Loss (%)			
	T1	T2	T3	T4	I	II	III	IV
**2-1**	88.7	198.8	292.6	418	1.9	19.8	43.7	34.6
**3-1**	87.9	199.6	301	408.6	2.3	16.7	48.4	32.7
**5-1**	83.3	204.6	293.1	373.5	3.8	13.9	54.2	28.1
**7-1**	91.1	205.7	291.2	369.8	3.8	13.6	60	22.7
**MDGA**	92.39	-	290.1	370.4	5.4	13.8	60.5	20.3
**AA**	-	230.8	312.2	498.5	0	33.3	26.3	40.4
**MD**	86.7	-	312.3	508.1	7.2	1.6	58	33.2
**GA**	79.4	-	284	430.5	12.5	3.3	51.9	32.3

## Data Availability

Data are contained within the article and Supplementary Materials.

## Supplementary Materials

The following supporting information can be downloaded at: https://www.mdpi.com/article/10.3390/molecules29153587/s1, Figure S1: Example of UV/vis spectrum of MDGA-AA particles after dissolution in acetate buffer (pH 5). Concentration of ascorbic acid (AA) is determined at 265 nm; Table S1: Particle size distribution and polydispersity index of MDGA-AA particles depending on wall material/active (WM:A) ratio (*w*/*w*). Data are shown as volume diameters; Figure S2: Evolution of sample’s weight for 9 weeks at 40 °C 90%RH; Figure S3: Chromatogram (265 nm) of pure AA and MDGA-AA particles after 1 year and 9 months at 4 °C and 40 °C, combined with negative-ion mode ESI mass spectroscopy.
