# Supplementary material for: Impact of Wall Material-to-Active Ratio in the Stability of Spray-Dried Ascorbic Acid Using Maltodextrin and Gum Arabic
Source: Molecules. 2024 Jul 30;29(15):3587. doi: 10.3390/molecules29153587 (PMC11314086; doi:10.3390/molecules29153587)
Supplement: Supplementary file 1 [file molecules-29-03587-s001.zip › molecules-3036148-supplementary.pdf]

Supplementary materials

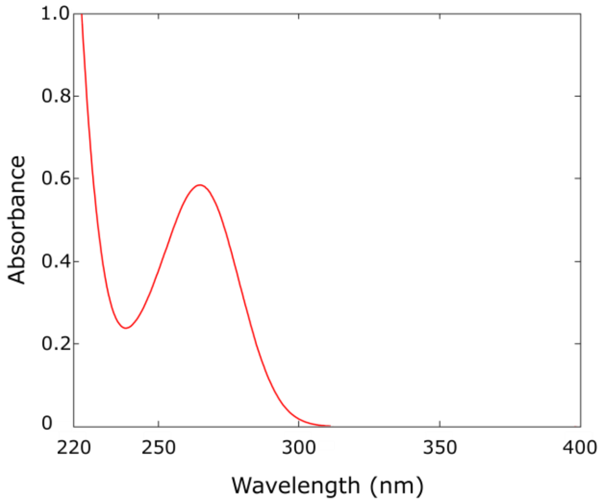

Figure S1 Example of UV-vis spectrum of MDGA-AA particles after dissolution in acetate buffer (pH 5). Concentration of ascorbic acid (AA) is determined at 265 nm.

Table S1 Particle size distribution and polydispersity index of MDGA-AA particles depending on wall material:active (WM:A) ratio (w/w). Data are shown as volume diameters.

Ratio WM:A	D[10] (µm)	D[50] (µm)	D[90] (µm)	SPAN
2:1	6.7 ± 2.6[a]	21.7 ± 10.9[a]	56.7 ± 43.8[a]	2.1 ± 0.6[a]
3:1	3.3 ± 1.3[b]	12.0 ± 4.6[b]	30.4 ± 19.3[ab]	2.1 ± 0.5[a]
5:1	3.2 ± 0.2[b]	10.2 ± 1.3[b]	23.9 ± 7.2[b]	2.0 ± 0.4[a]
7:1	2.3 ± 0.7[b]	7.1 ± 3.0[b]	19.2 ± 5.3[b]	2.9 ± 1.5[a]

[a,b,c,d] values with different letters in the same columns differ significantly ($p<0.05$).

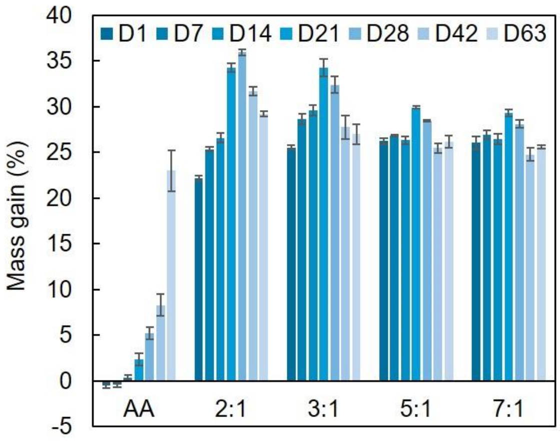

Figure S2 Evolution of sample's weight for 9 weeks at 40°C 90%RH

LC-MS Analyses

Sample preparation

Pure L-ascorbic acid (AA) and MDGA-AA microparticles (stored for 1 year and 9 months at 4°C and 40°C) were solubilized at 2 g/L and 10 g/L, respectively, in distilled water under magnetic stirring for 5 min. The solution was then filtered using a 0.45 µm pore-sized cellulose acetate filter.

Separation conditions (HPLC)

The concentration of AA in samples was measured using an LC-MS Agilent 1200 (Agilent Technologies, Waldbronn, Germany) equipped with a column Ascentis®Express (Supelco) RP-Amide, 2µm 10 cm x 2.1 mm column, and a UV/VIS detector Agilent Infinity 1260 VL+. The compound was eluted in a mobile phase consisting of 15% milliQ water and 85% acetonitrile in isocratic mode at a flow rate of 0.5 mL/min. The column temperature was maintained at 25 °C, and the detection wavelength was 265 nm. The AA elution time was 0.5 min.

Ionization conditions (mass spectroscopy)

Mass spectroscopy (MS) was performed on a mass spectrometer, Agilent Ion Trap 6340 (Agilent Technologies, Waldbronn, Germany), monitored by Agilent Ion Trap software (for acquisition and analysis). Samples were introduced into the MS via the HPLC stream. The MS conditions were negative electrospray ionization (ESI) mode with nebulizer pressure at 15 psi. Nitrogen was used as the drying gas under a flow rate of 5 L/min and a temperature of 350 C. The acquisition was performed between 100 and 200 m/z.

HPLC analysis of MDGA-AA microparticles

To validate the presence of degradation products, MDGA-AA particles maintained at 4°C and 40°C for 1 year and 9 months (1Y9M) were examined using HPLC-MS. As expected, AA was the only visible component at 265 nm (at 0.5 min) and was confirmed by ESI mass spectroscopy, which revealed a m/z of 174.8, corresponding to the ion ascorbate. In conclusion, HPLC and UV-vis measurements yield the same results.

.

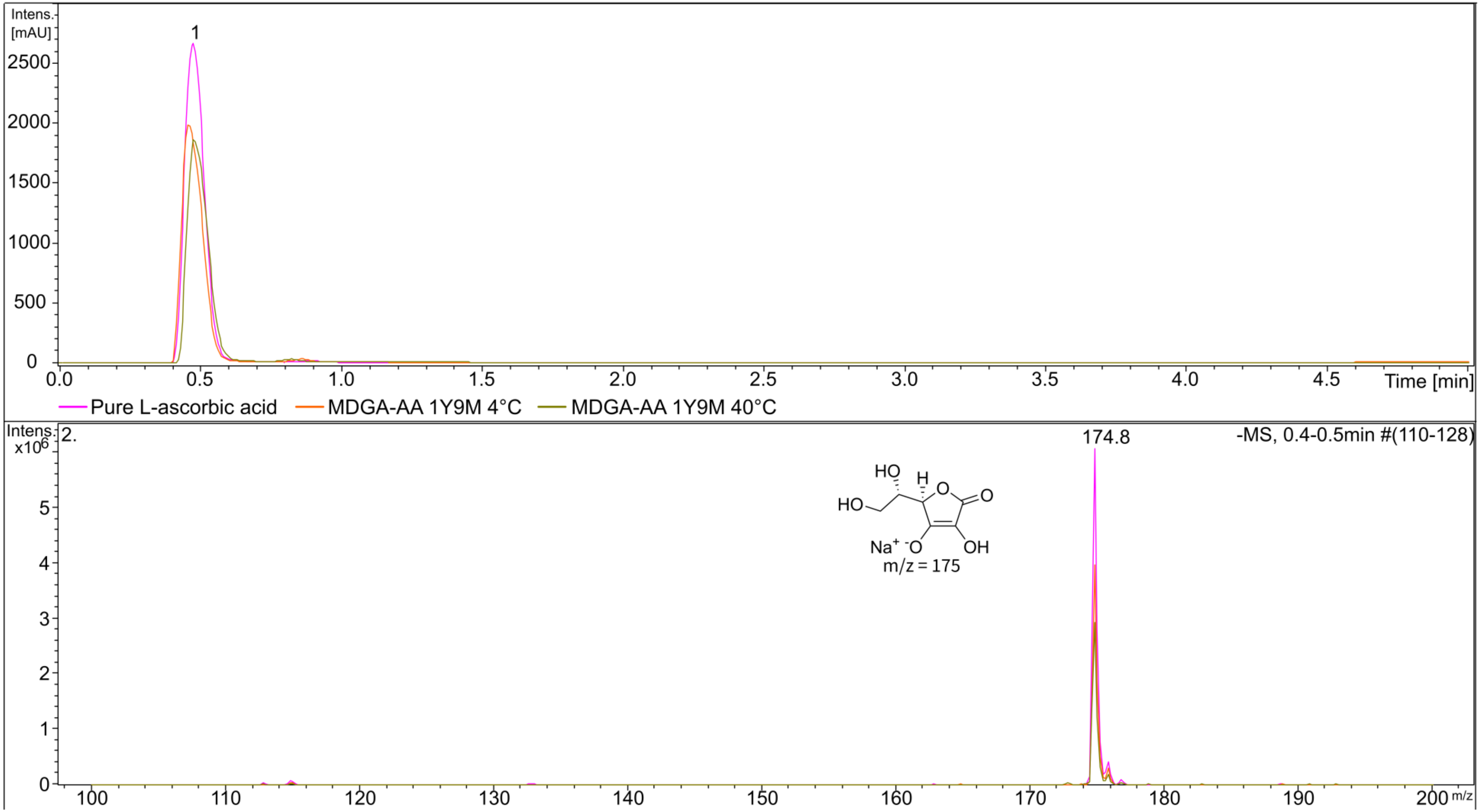

Figure S3 Chromatogram (265 nm) of pure AA and MDGA-AA particles after 1 year and 9 months at 4°C and 40°C, combined with negative ion mode ESI mass spectroscopy.
